# Relationship between the Renal Function and Adverse Clinical Events in Patients with Atrial Fibrillation: A Japanese Multicenter Registry Substudy

**DOI:** 10.3390/jcm9010167

**Published:** 2020-01-08

**Authors:** Yasuhumi Yuzawa, Keiichiro Kuronuma, Yasuo Okumura, Katsuaki Yokoyama, Naoya Matsumoto, Eizo Tachibana, Koji Oiwa, Michiaki Matsumoto, Toshiaki Kojima, Hironori Haruta, Kazumiki Nomoto, Kazumasa Sonoda, Ken Arima, Rikitake Kogawa, Fumiyuki Takahashi, Tomobumi Kotani, Kimie Okubo, Seiji Fukushima, Satoru Itou, Kunio Kondo, Masaaki Chiku, Yasumi Ohno, Motoyuki Onikura, Atsushi Hirayama

**Affiliations:** 1 Department of Cardiology, Nihon University Hospital, Tokyo 101-8309, Japan; 2 Kawaguchi Municipal Medical Center, Kawaguchi 333-0833, Japan; 3 Division of Cardiology, Nihon University Itabashi Hospital, Tokyo 173-8610, Japan; 4 Yokohama Chuo Hospital, Yokohama 231-0023, Japan; 5 Sekishindo Hospital, Kawagoe 350-1123, Japan; 6 Asaka Medical Center, Asaka 351-0023, Japan; 7 Tokyo Rinkai Hospital, Tokyo 134-0086, Japan; 8 Kasukabe Municipal Hospital, Kasukabe 344-8588, Japan; 9 Yasuda Hospital, Tokyo 175-0094, Japan; 10 Makita General Hospital, Tokyo 143-0016, Japan; 11 Itabashi Medical Association Hospital, Tokyo 175-0082, Japan; 12 Ukima Central Hospital, Tokyo 115-0052, Japan; 13 Itou Cardiovascular Clinic, Tokorozawa 359-1124, Japan; 14 Kondo Clinic, Tokyo 167-0022, Japan; 15 Keiai Clinic, Tokyo 173-0036, Japan; 16 Ohno Medical Clinic, Tokyo 173-0004, Japan; 17 Onikura Clinic, Yachiyo 276-0023, Japan

**Keywords:** adverse clinical events, atrial fibrillation, direct oral anticoagulant, Japanese, renal function

## Abstract

Background: Atrial fibrillation (AF) and chronic kidney disease (CKD) often coexist, but the real-world data after approval of direct oral anticoagulants (DOACs) are still lacking in Japan. We investigated the association of the baseline renal function and adverse clinical events and risk of adverse clinical events with DOACs compared to warfarin for each renal functional level in Japanese AF patients. Methods: The present substudy was based on the SAKURA AF Registry, a Japanese multicenter observational registry (median follow-up period: 39 months). The creatinine clearance (CrCl) values were estimated by the Cockcroft–Gault formula, and divided into normal renal function, and mild and moderate-severe CKD (CrCl ≥ 80, 50–79, <50 mL/min). Results: In the SAKURA AF Registry, the baseline CrCl data were available for 3242 patients (52% for DOAC and 48% for warfarin user). The relative risk of adverse clinical events was significantly higher in the patients with a CrCl < 50 mL/min as compared to those with a CrCl ≥ 80 mL/min (adjusted HRs: 2.53 for death, 2.53 for cardiovascular [CV] events, 2.13 for strokes, and 1.83 for major bleeding). Risks of all adverse clinical events were statistically even between DOAC and warfarin users for each renal function level. Conclusion: Moderate–severe CKD was associated with a higher mortality, CV events, strokes, and major bleeding than normal renal function. The safety and effectiveness of DOACs over warfarin were similar for each renal function level. By a worsening renal function, the incidence of adverse clinical events increased, especially deaths and CV events as compared to strokes and major bleeding.

## 1. Introduction

Atrial fibrillation (AF) and chronic kidney disease (CKD) are common cardiac conditions that often coexist and predispose to each other [1,2]. Since 2007, Japan has been a super-aging society, and accordingly, the incidence of AF is increasing. The expected number of AF patients will be over one million by 2030 in Japan [3]. A prior study showed that approximately 65% of AF patients were shown to have mild-to-severe CKD (estimated glomerular filtration rate (eGFR) < 80 mL/min/1.73 m^2^) [4]. For stroke prevention in AF patients, we generally recommend anticoagulation therapy, which is with direct oral anticoagulants (DOACs) or vitamin K antagonists (e.g., warfarin). There are four DOACs including dabigatran, rivaroxaban, apixaban, and edoxaban, and large randomized controlled trials (RCTs) have shown the non-inferiority or superiority of DOACs compared to warfarin in the light of the safety and efficacy [5,6,7,8]. Due to the convenience of DOACs over warfarin, such as the needlessness of anticoagulation monitoring and less food interactions, DOACs are widely used in clinical settings instead of the conventional anticoagulant treatment, warfarin. Huiart et al. reported that 61% of AF patients initially used one of the four DOACs as their anticoagulation therapy [9].

AF and CKD are widely known as risk factors for adverse clinical events (e.g., strokes, major bleeding, and death). The substudy of the ATRIA study showed that AF patients with renal dysfunction had a higher rate of thromboembolisms than those with normal renal function [10]. When we focused on Japanese cohort studies, the analyses related to renal function were performed in the J-RHYTHM Registry [11] and Fushimi AF Registry [12]. Those registries also showed that AF patients with renal impairment were associated with adverse clinical events, however, the rates of DOAC use were none or less than 10% because those registries were performed before or soon after DOACs became approved in Japan. In the Japanese AF patients stratified by the baseline renal function, the safety and effectiveness of DOACs as compared to warfarin were not evaluated in a Japanese registry.

By using the data from the SAKURA AF registry, a Japanese multicenter observational registry that included 3267 AF patients and over 50% being DOAC users (median follow-up period: 39 months), the present study was undertaken to analyze the clinical outcomes between the patients with and without renal dysfunction at baseline, and those between the patients taking DOACs and warfarin at each renal function level.

## 2. Methods

### 2.1. Study Design

Our investigation was conducted as a sub-study of the SAKURA AF Registry (UMIN000014420) [13,14], which was set up to support multicenter, prospective observational research by tracking clinical events in patients with AF for at least 2 years and up to 4 years after their enrollment in the registry. A total of 3267 patients were enrolled from 63 institutions (2 cardiovascular centers, 20 affiliated hospitals or community hospitals, and 41 private clinics) in the Tokyo area between September 2013 and December 2015 (Acknowledgments). The patients were eligible for inclusion if they were ≥20 years old, had been diagnosed as having non-valvular AF, and were being initiated/had already been initiated on oral anticoagulant therapy for stroke prevention. All the patients provided written informed consent for inclusion in the Registry. 

### 2.2. Data Correction and Outcome Measures

The patient data for the Registry were collected by means of a website and web-based registration system created for the Registry, through which, relevant clinical data such as the body height, body weight, type of AF (paroxysmal or non-paroxysmal AF), medical history, and laboratory data, including the prothrombin time-international normalized ratio (PT-INR) in warfarin users, and the serum creatinine (SCr) concentration, were collected. Follow-up information including those laboratory data were entered twice a year. Paroxysmal AF was defined as AF lasting for up to 7 days. A history of heart failure was defined as a diagnosis of heart failure prior to enrollment of the patient in the Registry. New use of an oral anticoagulant (OAC), defined as an OAC therapy initiated within 3 months before the patient’s enrollment, was noted. The time in a therapeutic range (TTR) was calculated by the Rosendaal method [15]. We calculated the TTR assuming a PT-INR of 1.6–2.6 for those aged ≥70 years and a PT-INR of 2.0–3.0 for those aged <70 years, according to the 2013 Japanese Circulation Society guidelines [16]. Good PT-INR control was defined arbitrarily as a TTR ≥65%. The creatinine clearance (CrCl) was estimated by the Cockcroft–Gault formula [17]: CrCl (mL/min) = (140 − age (years)) × (weight (kg))/(72 × SCr (mg/dL)) × 0.85 (if female). For the purpose of this study, we defined a normal renal function as a CrCl greater than or equal to 80 mL/min, mild CKD as a CrCl between 50 and 80 mL/min, and moderate-to-severe CKD as a CrCl less than 50 mL/min. This cutoff value for the renal function was used in previous large trial analyses [18,19]. We also recorded the incidences of death from any cause, cardiovascular events, strokes (ischemic strokes, hemorrhagic strokes, or transient ischemic attacks (TIAs))/systemic embolisms (SEs), and major bleeding. Cardiovascular (CV) events included the following events; heart failure requiring hospital admission, myocardial infarction, unstable angina, other cardiovascular events requiring hospital admission, and cardiac death. Strokes/SEs were excluded from the CV events. Major bleeding was defined as a reduction in the blood hemoglobin concentration by at least 2 g/dL, the need for a blood transfusion of at least 2 units of blood, and/or symptomatic bleeding in any critical area or organ. An analysis of the data from the Registry was conducted with the approval of the Nihon University School of Medicine Itabashi Hospital institutional review board and the review boards of the hospitals at which the patients were receiving treatment.

### 2.3. Statistical Analysis

The categorical variables are shown as numbers and percentages and continuous variables as the mean ± standard deviation or median values (25th, 75th percentiles), as appropriate. Differences in the categorical variables among the renal function groups were analyzed by a Fisher’s exact test, while those for the continuous variables were analyzed by an analysis of variance or Kruskal–Wallis test, as appropriate. The trend tests were performed by using the Cochran–Armitage trend test for categorical variables and Jonckheere–Terpstra trend test for continuous variables. Kaplan–Meier curves were drawn for the time-to-events, which were compared by the log-rank test. To examine the associations between the renal dysfunction levels and development of adverse clinical events, we divided the subjects into 3 groups using the CrCl values; normal renal function (CrCl ≥ 80 mL/min), mild CKD (CrCl 50–79 mL/min), and moderate-severe CKD (CrCl < 50 mL/min). A Cox proportional hazards model was used to assess the associations of the renal function levels with the risk of adverse clinical events, and the results are shown in terms of the hazard ratios (HRs) and 95% confidence intervals (CIs). For the multivariable analysis, we used the following factors as explanatory variables: the components of the CHADS_2_ score (congestive heart failure, hypertension, age ≥ 75 years, diabetes mellitus, and a prior stroke or TIA) [20], sex category, body weight (<50 kg), type of AF, history of AF ablation, DOAC use, and antiplatelet drug use. The values of the age, body weight, and CrCl were also introduced into the model as continuous variables. In this case, the CrCl values were not normally distributed, and therefore, the log CrCl was used for the model. In the Cox model, we also assessed the consistency of the treatment effects by testing for any interactions between the treatment strategy (DOAC vs. warfarin) and renal function groups. All statistical analyses were performed with the JMP 14.1.0 (SAS Institute Inc., Cary, NC, USA) or EZR software (Saitama Medical Center, Jichi Medical University, Saitama, Japan), which is a graphical user interface for R (The R Foundation for Statistical Computing, Vienna, Austria) [21]. A two-sided *p* value of <0.05 was considered as being indicative of statistical significance.

## 3. Results

### 3.1. Baseline Characteristics

Because of the lack of SCr data, the baseline CrCl could be estimated in 3242 out of the total 3267 enrollees enrolled in this study. Of the 3242 total patients, 1679 (51.8%) were DOAC users and 1563 (48.2%) warfarin users. The median CrCl was 64.65 (IQR 50.16–82.03) mL/min. The number of patients in each group was 893 in the normal renal function group, 1550 in the mild CKD group, and 799 in the moderate-to-severe CKD group. Of those patients, the one-year follow-up information was entered for 3157 patients (97.4%) and two-year follow-up data for 2952 (91.1%). The baseline characteristics of the overall subject population and of each of the CrCl groups are shown in Table 1. Compared to the patients with a CrCl ≥ 80 mL/min, the patients with a CrCl < 50 mL/min were older, more frequently were female, had a lower body weight, were more likely to have non-paroxysmal AF, and to have higher CHADS_2_ and CHA_2_DS_2_-VASc scores. The prevalence of hypertension and diabetes did not significantly differ between the three groups.

### 3.2. Clinical Outcomes

**Figure 1:**
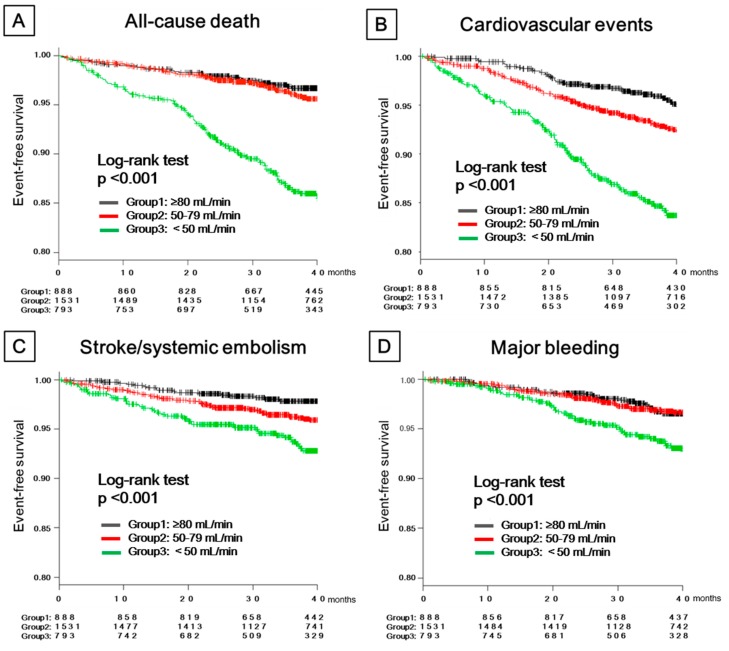
Kaplan–Meier curves for all-cause death, cardiovascular events, strokes/systemic embolisms, and major bleeding in patients classified by their renal function: (**A**) Kaplan–Meier curves of all-cause death, (**B**) Kaplan–Meier curves of cardiovascular events, (**C**) Kaplan–Meier curves of stroke/systemic embolism, and (**D**) Kaplan–Meier curves of major bleeding.

The median follow-up period was 39.4 (IQR 28.7–43.6) months, and the total person-years of this cohort was 9606. During the follow-up period, 198 patients (2.1 per 100 person-years) died; CV events occurred in 264 patients (2.8 per 100 person-years); strokes/SEs occurred in 131 patients (1.4 per 100 person-years); and major bleeding occurred in 123 patients (1.3 per 100 person-years). CV events were attributed to heart failure (*n* = 144 (54.5%)), cardiac death (*n* = 46 (17.4%)), myocardial infarctions and unstable angina (*n* = 21 (8.0%)), and other cardiovascular events (*n* = 53 (20.1%)). Kaplan–Meier curves for these adverse clinical events were drawn for each of the CrCl groups and are shown in Figure 1. 

The patients with a low CrCl at the time of enrollment had a low event-free survival rate for all adverse clinical events in this study. The results of the Cox proportional hazard analysis related to the renal function are shown in Table 2. 

The risk of death was shown to be significantly higher for patients with a CrCl < 50 mL/min than in those with a CrCl ≥ 80 mL/min (adjusted HR 2.40, 95% CI 1.41–4.07; *p* < 0.0001), as was the risk of CV events (adjusted HR 2.53, 95% CI 1.62–3.94; *p* < 0.0001), stroke/SE events (adjusted HR 2.13, 95% CI 1.34–4.00; *p* = 0.0182), and major bleeding events (adjusted HR 1.83, 95% CI 1.02–3.29; *p* = 0.0434). The results of the multivariable analysis of all factors used for the adjustment are shown in the Table S1. Besides the impaired renal function, the age (≥75 years) (adjusted HR 2.59, 95% CI 1.78–3.75; *p* < 0.0001) and a female sex (adjusted HR 0.57, 95% CI 0.39–0.84; *p* = 0.0038) were significantly associated with death, the age (≥75 years) (adjusted HR 1.45, 95% CI 1.09–1.93; *p* = 0.0117), paroxysmal AF (adjusted HR 1.35, 95% CI 1.03–1.76; *p* = 0.0269), history of heart failure (adjusted HR 2.10, 95% CI 1.62–2.74; *p* < 0.0001), and antiplatelet use (adjusted HR 1.78, 95% CI 1.35–2.36; *p* < 0.0001) were associated with CV events, and a history of a stroke/TIA (adjusted HR 2.17, 95% CI 1.44–3.27; *p* = 0.0002) was correlated with a stroke/SE. Only an impaired renal function was significantly associated with major bleeding (Table S1). When assessing the continuous variables, the log CrCl was associated with death (adjusted HR 0.49, 95% CI 0.31–0.76; *p* = 0.0017), CV events (adjusted HR 0.41, 95% CI 0.29–0.58; *p* < 0.0001), stroke/SE events (adjusted HR 0.50, 95% CI 0.28–0.88; *p* = 0.0171), and major bleeding events (adjusted HR 0.43, 95% CI 0.25–0.75; *p* = 0.0029). The results of this analysis of all factors are shown in Table S2.

Using a Cox proportional hazard analysis, in each renal function level, the risk of all adverse events did not significantly differ between the DOAC and warfarin users (Table 3). 

**Figure 2:**
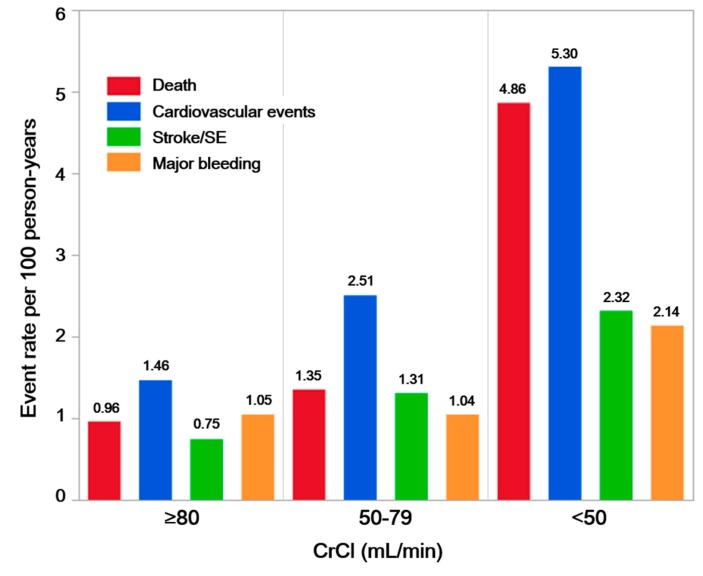
Event rate per 100 patient-years of adverse clinical events for each renal function stratified by the creatinine clearance at study entry.

The results of the incident number (parson-year) for each adverse clinical event in each CrCl group are shown in Figure 2. In the group of patients with a CrCl ≥ 80 mL/min, each adverse clinical event rate was shown to be almost equal (0.75–1.46 per 100 person-years). By a worsening renal function, the incidence of each adverse event increased, and especially, increasing rates of death and CV events were high. In the group of patients with a CrCl < 50 mL/min, the clinical event rates were as follows: 4.86, 5.30, 2.32, and 2.14 per 100 person-years, for deaths, CV events, strokes/SEs, and major bleeding, respectively.

## 4. Discussion

There were three major findings of this study: (1) patients with moderate-severe CKD had a higher rate of all adverse clinical events including strokes/SEs, major bleeding, CV events, and death, as compared to those with a normal renal function. These results remained even after an adjustment by a multivariable analysis, (2) the use of DOACs (vs. warfarin) was not associated with a risk reduction of adverse clinical events, and (3) all adverse clinical event rates increased with a worsening renal function. The increased gradient of the death and CV event rates was predominant as compared to that of strokes/SEs and major bleeding.

The SAKURA AF Registry is a large-scale registry designed to collect data that can be used for a prospective evaluation of the outcomes among AF patients in Japan who are treated with DOACs or warfarin, and eventually, the usage of each OAC were found to be equal (51.8% for DOACs use and 48.2% for warfarin use). Since 2011, DOACs have been approved in Japan, and their prescription has been increasing because of the safety and convenience of DOACs compared to warfarin. In the SAKURA AF registry, DOAC users increased from 51.8% at baseline to 54.9% at two-years of follow-up [14]. Because the previous Japanese registries were performed previous to the present study, the usage of DOACs in the previous registries was much lower than that in the present study (none for the J-RHYTHM Registry [22], and 7.2% for the FUSHIMI AF Registry [23]). We think, therefore, that the present registry is a better reflection of the current status of AF treatment in Japan, but the clinical use of DOACs are dramatically increasing now. The prevalence of each baseline renal function level in our registry was 27.5% for a CrCl ≥ 80 mL/min, 47.8% for a CrCl 50–79 mL/min, and 24.6% for a CrCl < 50 mL/min. That was quite similar to the J-RHYTHM Registry, which used the Cockcroft-Gault formula to calculate the CrCl (29.9% for a CrCl ≥ 80 mL/min, 44.3% for a CrCl 50–79 mL/min, and 25.7% for a CrCl < 50 mL/min) [11]. One of the major findings of our registry was that patients with a CrCl of < 50 mL/min had high HRs for all adverse events than did those with a normal renal function and its significance remained after a multivariable analysis (adjusted HR (95% CI): 2.40 (1.41–4.07) for death, 2.53 (1.62–3.94) for CV events, 2.13 (1.34–4.00) for strokes/SEs, and 1.83 (1.02–3.29) for major bleeding). To stratify the AF patients in terms of the risk of thromboembolisms or major bleeding, we often check the CHADS_2_ score or HAS-BLED score [20,24]. In the present study, a multivariable analysis showed that only an impaired renal function was significantly associated with all adverse events, and the relative risk of all events was almost the highest among the other variables such as the age or comorbidities (Table S1). Those results were also consistent when the CrCl values were assessed as continuous values. Therefore, an impaired renal function (CrCl < 50 mL/min) would simply and accurately predict the adverse events in AF patients taking an OAC therapy. Compared to the other Japanese registry, the results of the adjusted HR for major bleeding events differed from that of the J-RHYTHM Registry [11], in which the adjusted HRs (95% CI) for major hemorrhage events were not significant, 1.10 (0.58–2.09) for a CrCl 30–50 mL/min, and 1.37 (0.62–3.03) for a CrCl < 30 mL/min (vs. CrCl ≥ 80 mL/min). In the Fushimi AF Registry, the adjusted HR for major bleeding was 2.08 (1.23–3.39) for a CrCl < 30 mL/min (vs. CrCl ≥ 50 mL/min). The reason for this difference may be the difference in the patient’s characteristics, status of the anticoagulated therapy, and definition of major bleeding events (major hemorrhage requiring hospital admission in the J-RHYTHM Registry [25], and the same definition in the present study and Fushimi AF Registry [12]).

In the present study, we analyzed the safety and effectiveness of DOACs as compared to warfarin in each renal function group, and those were statistically even between the DOACs and warfarin (Table 3). In a previous subanalysis of the RCTs regarding renal function, the safety and efficacy of DOACs (vs. warfarin) were statistically shown. For major bleeding events, a low adjusted HR of apixaban compared to warfarin was shown in patients with a CrCl < 50 mL/min (HR; 0.50, 95% CI; 0.38–0.66) [18] and for that of edoxaban in patients with a CrCl < 50 mL/min (HR; 0.76, 95% CI; 0.58–0.98) [26]. For stroke/SE events, a low adjusted HR of dabigatran compared to warfarin was shown in patients with a CrCl < 50 mL/min (HR; 0.56, 95% CI; 0.37–0.85) and for that of patients with a CrCl 50–79 mL/min (HR; 0.68, 95% CI; 0.50–0.92) [19]. Compared to the present study, those RCTs included high risk patients with a CHADS_2_ score of more than 1–2 points and a high rate of antiplatelet therapy (29%–40%) [5,6,7,8]. In addition, the median TTR of the present study was 71.5%, and 95% of warfarin users had already begun warfarin therapy for over 3 months at the time of enrollment [14]. These patient background differences, and stability of the warfarin therapy may have attributed to the equality of the clinical outcomes between DOACs and warfarin in the present Japanese real-world registry. This finding supports that warfarin users with a stable PT-INR control could safely and effectively continue warfarin therapy as compared to DOACs, regardless of having concomitant CKD. 

The incidence rates of each adverse event increased with a worsening renal function at baseline (Figure 2). The incidence rates of each adverse event were equal in the patients with a normal renal function, and furthermore, those of death and CV events were more than two-fold compared to those for strokes/SEs and major bleeding in the patient group with a CrCl < 50 mL/min. In the large RCTs comparing DOACs versus warfarin, the all-cause mortality was 2.04%–2.71%/year in patients with a normal renal function, 3.15%–3.56%/year in patients with mild CKD, and 6.28%–8.30%/year in patients with moderate-severe CKD. The annual stroke/SE rate was 0.81%–1.12%/year in patients with a normal renal function, 1.21%–1.69%/year in patients with mild CKD, and 1.21%–2.67%/year in patients with moderate-severe CKD. The incidence rate of major bleeding was 1.25%–1.84%/year in patients with a normal renal function, 2.45%–3.21%/year in patients with mild CKD, and 3.21%–6.44%/year in patients with moderate–severe CKD [18,19]. In the prospective GARFIELD-AF Registry including 35 countries and over 33,000 patients, the events rates per 100 person-years stratified by the renal function were as follows: deaths: 3.22 for a normal renal function, 5.60 for mild CKD, and 10.35 for moderate-severe CKD; strokes/SEs: 1.03 for a normal renal function, 1.21 for mild CKD, and 2.25 for moderate-severe CKD; and major bleeding: 0.63 for a normal renal function, 0.98 for mild CKD, and 1.85 for moderate-severe CKD) [27]. Although the adverse event rates in the present Japanese study were lower than those in the previous worldwide data, the tendency of the event rates in each renal function group was similar to the present study. This finding suggested that when examining AF patients, we should understand that the characteristics of possible adverse events vary with the status of the renal function, and patients with AF and an impaired renal function should be more aware of deaths and CV events as well as strokes/SEs and major bleeding.

Our study results should be interpreted in light of the study limitations. First, the study was conducted as a retrospective analysis of prospectively collected data. Some findings were not consistent with those of prior RCTs, however, we believe that the present real-world registry data reflects the current status of AF treatment in Japan, and has a clinical implication for the clinical management of AF patients receiving OACs. Second, we were not able to collect the renal function data for all participants by using the Cockcroft–Gault formula. Third, in the present study, the number of users of each of the four DOACs was not sufficient to allow for a multivariate outcome analysis. Finally, only selected institutions within a limited geographical area in Japan participated in the SAKURA AF Registry, and it would be difficult to assume that our findings can be generalized to the entire population of AF patients in Japan. It should be noted, however, that patient selection and regional enrollment biases are limitations of all prospective observational studies.

## 5. Conclusions

Patients with moderate-severe CKD had a high risk for death, CV events, strokes/SEs, and major bleeding. For each renal function level, a superiority of DOACs over warfarin regarding the clinical outcomes was not found. We should understand that the trend in the adverse clinical outcomes in AF patients seems to differ according to the baseline renal function level.

## Figures and Tables

**Table 1:** Patient characteristics according to the renal function.

Number of Patients (%)	Overall	Normal Renal Function (CrCl ≥ 80 mL/min)	Mild CKD (CrCl 50–79 mL/min)	Moderate–Severe CKD (CrCl < 50 mL/min)	*p*-Value *	*p*-Value for Trend
	3242	893 (27.5)	1550 (47.8)	799 (24.6)		
Age (years)	72.00 ± 9.38	63.54 ± 8.70	72.82 ± 6.61	79.87 ± 6.69	<0.001	<0.001
<65	614 (18.9)	443 (49.6)	159 (10.3)	12 (1.5)	<0.001	<0.001
65–74	1286 (39.7)	376 (42.1)	759 (49.0)	151 (18.9)		
≥75	1342 (41.4)	74 (8.3)	632 (40.8)	636 (79.6)		
Female sex	848 (26.2)	115 (12.9)	410 (26.5)	323 (40.4)	<0.001	<0.001
Body height (cm)	162.48 ± 9.49	168.01 ± 7.96	162.31 ± 8.41	156.62 ± 9.44	<0.001	<0.001
Body weight (kg)	63.85 ± 12.96	74.16 ± 12.47	62.67 ± 10.09	54.61 ± 10.17	<0.001	<0.001
BMI (kg/m^2^)	24.05 ± 3.73	26.25 ± 4.01	23.74 ± 3.16	22.19 ± 3.19	<0.001	<0.001
Paroxysmal AF	1195 (36.9)	351 (39.3)	593 (38.3)	251 (31.4)	0.001	0.001
Medical history						
Hypertension	2312 (71.3)	616 (69.0)	1106 (71.4)	590 (73.8)	0.087	0.027
Dyslipidemia	1256 (38.7)	371 (41.5)	635 (41.0)	250 (31.3)	<0.001	<0.001
Diabetes	741 (22.9)	221 (24.7)	338 (21.8)	182 (22.8)	0.249	0.310
Heart failure	719 (22.2)	165 (18.5)	292 (18.8)	262 (32.8)	<0.001	<0.001
Stroke/TIA	364 (11.2)	54 (6.0)	193 (12.5)	117 (14.6)	<0.001	<0.001
Ischemic heart disease	312 (9.6)	57 (6.4)	159 (10.3)	96 (12.0)	<0.001	<0.001
AF ablation	299 (9.2)	147 (16.5)	123 (7.9)	29 (3.6)	<0.001	<0.001
DOAC use	1679 (51.8)	493 (55.2)	818 (52.8)	368 (46.1)	<0.001	<0.001
Warfarin use	1563 (48.2)	400 (44.8)	732 (47.2)	431 (53.9)	<0.001	<0.001
TTR (%)	71.50 (43.20, 93.40)	64.60 (33.80, 87.67)	74.60 (46.18, 94.30)	74.20 (49.70, 94.90)	<0.001	<0.001
TTR ≥ 65%	805 (57.6)	176 (49.7)	394 (60.1)	235 (60.7)	0.002	0.003
Antiplatelet use	517 (15.9)	92 (10.3)	248 (16.0)	177 (22.2)	<0.001	<0.001
Antiarrhythmic drug class Ⅰ	423 (13.0)	141 (15.8)	196 (12.6)	86 (10.8)	0.007	0.002
Beta-blocker use	1471 (45.4)	403 (45.1)	689 (44.5)	379 (47.4)	0.382	0.362
Amiodarone use	32 (1.0)	9 (1.0)	11 (0.7)	12 (1.5)	0.184	0.334
Bepridil use	322 (9.9)	113 (12.7)	168 (10.8)	41 (5.1)	<0.001	<0.001
CHADS_2_ score	2 (1, 2)	1 (1, 2)	2 (1, 2)	2 (2, 3)	<0.001	<0.001
CHA_2_DS_2_-VASc score	3 (2, 4)	2 (1, 3)	3 (2, 4)	4 (3, 5)	<0.001	<0.001
New use (OAC therapy duration <3 months)	637 (19.6)	186 (20.8)	301 (19.4)	150 (18.8)	0.541	0.283
SCr (mg/dL)	0.87 (0.75, 1.04)	0.78 (0.69, 0.86)	0.88 (0.75, 1.00)	1.09 (0.88, 1.34)	<0.001	<0.001
CrCl (mL/min)	64.65 (50.16, 82.03)	94.26 (86.11, 108.90)	63.74 (57.01, 70.88)	40.05 (32.57, 45.93)	<0.001	<0.001

Values are the mean ± SD, median (25th, 75th percentiles), or number (%) of patients. AF = atrial fibrillation; BMI = body mass index; CHADS_2_ = congestive heart failure, hypertension, age > 75 years, diabetes mellitus, prior stroke or TIA (doubled); CHA_2_DS_2_-VASc, congestive heart failure, hypertension, age >75 years (doubled), Diabetes mellitus, prior stroke, TIA, or thromboembolic event (doubled), vascular disease, age 65–74 years, sex category; CKD = chronic kidney disease; CrCl = creatinine clearance; DOAC = direct oral anticoagulant; OAC = oral anticoagulant; SCr = serum creatinine; TIA = transient ischemic attack; TTR = time therapeutic range. * *p*-Value for comparison between renal function groups based on Fisher’s exact test, analysis of variance, or Kruskal–Wallis test, as appropriate.

**Table 2:** Adverse clinical outcomes and results of the Cox proportional hazards model according to the renal function.

Outcome	Number of Patients	Number of Events	Hazard Ratio			
CrCl (mL/min)			Crude (95% CI)	*p*-Value	Adjusted (95% CI)	*p*-Value
Death						
≥80 (reference)	893	26	1.00		1.00	
50–79	1550	63	1.41 (0.89–2.23)	0.138	0.99 (0.60–1.62)	0.9635
<50	799	109	5.14 (3.35–7.89)	<0.0001	2.40 (1.41–4.07)	0.0012
CV events						
≥80 (reference)	893	39	1.00		1.00	
50–79	1550	113	1.72 (1.19–2.47)	0.0036	1.51 (1.03–2.22)	0.0358
<50	799	112	3.67 (2.55–5.28)	<0.0001	2.53 (1.62–3.94)	<0.0001
Stroke/SE						
≥80 (reference)	893	20	1.00		1.00	
50–79	1550	60	1.77 (1.07–2.93)	0.0273	1.45 (0.84–2.47)	0.1787
<50	799	51	3.15 (1.88–5.28)	<0.0001	2.13 (1.34–4.00)	0.0182
Major bleeding						
≥80 (reference)	893	28	1.00		1.00	
50–79	1550	48	1.00 (0.63–1.59)	0.9981	0.92 (0.56–1.51)	0.7389
<50	799	47	2.08 (1.30–3.33)	0.0021	1.83 (1.02–3.29)	0.0434

The Cox model was adjusted for the sex, age (≥75 years), lower body weight (<50 kg), AF type, hypertension, diabetes, history of heart failure, history of a stroke/TIA, history of AF ablation, DOAC use, antiplatelet use. CI = confidence intervals; CrCl = creatinine clearance; CV = cardiovascular; SE = systemic embolism.

**Table 3:** Adjusted hazard ratio of the adverse clinical events of DOACs (versus warfarin) stratified by the renal function.

Clinical Outcome	Normal Renal Function (CrCl ≥ 80 mL/min)		Mild CKD (CrCl 50–79 mL/min)		Moderate-Severe CKD (CrCl < 50 mL/min)		*p*-Value for Interaction
	Adjusted HR (95% CI)	*p*-Value	Adjusted HR (95% CI)	*p*-Value	Adjusted HR (95% CI)	*p*-Value	
Death	0.97 (0.44–2.12)	0.9112	1.09 (0.66–1.81)	0.7322	0.99 (0.67–1.47)	0.9671	0.9369
Cardiovascular events	1.26 (0.66–2.41)	0.4889	1.19 (0.81–1.73)	0.3721	1.02 (0.69–1.50)	0.9272	0.6535
Stroke/SE	0.77 (0.31–1.89)	0.5705	1.43 (0.85–2.41)	0.1774	1.30 (0.74–2.27)	0.3664	0.4442
Major bleeding	0.90 (0.42–1.91)	0.7825	0.98 (0.55–1.74)	0.9443	0.95 (0.53–1.73)	0.8775	0.9946

Adjusted for the sex, age (≥75 years), lower body weight (<50 kg), AF type, hypertension, diabetes, history of heart failure, history of a stroke/TIA, history of AF ablation, antiplatelet use. CI = confidence intervals; CKD = chronic kidney disease; CrCl = creatinine clearance; HR = hazard ration; SE = systemic embolism. *p*-Value for interaction is for the interaction of treatment and renal function subgroups.

## Supplementary Materials

The following are available online at https://www.mdpi.com/2077-0383/9/1/167/s1, Table S1: Multivariable analysis of the risk factors for each adverse clinical event, Table S2. Multivariable analysis of the risk factors for each adverse clinical event by using continuous variables for all explanatory factors.
