# Supplementary material for: Relationship between the Renal Function and Adverse Clinical Events in Patients with Atrial Fibrillation: A Japanese Multicenter Registry Substudy
Source: J Clin Med. 2020 Jan 8;9(1):167. doi: 10.3390/jcm9010167 (PMC7019418; doi:10.3390/jcm9010167)
Supplement: Supplementary file 1 [file jcm-09-00167-s001.zip › jcm-676206-SI.pdf]

Table S1. Multivariable analysis of the risk factors for each adverse clinical event.

Outcomes	Adjusted HR	95% CI min	95% CI max	p-Value
**Death**				
CrCl <50 mL/min (vs. CrCl ≥80mL/min)	2.40	1.41	4.07	0.0012*
Age ≥75 years	2.59	1.78	3.75	<.0001*
Female sex	0.57	0.39	0.84	0.0038*
Body weight (<50 kg)	1.29	0.85	1.96	0.2268
Paroxysmal AF (vs. non-paroxysmal AF)	0.83	0.60	1.15	0.2624
Hypertension	0.76	0.56	1.03	0.0763
Diabetes	1.20	0.87	1.65	0.2682
History of heart failure	0.83	0.61	1.15	0.2682
History of a stroke/TIA	1.01	0.66	1.54	0.9588
History of AF ablation	0.68	0.31	1.49	0.3375
DOAC use (vs. warfarin)	1.02	0.76	1.35	0.9106
Antiplatelet use	1.20	0.85	1.68	0.2999
**Cardiovascular events**				
CrCl <50 mL/min (vs. CrCl ≥80mL/min)	2.53	1.62	3.94	<.0001*
Age ≥75 years	1.45	1.09	1.93	0.0117*
Female sex	0.80	0.59	1.09	0.158
Body weight (<50 kg)	1.07	0.73	1.56	0.740
Paroxysmal AF (vs. non-paroxysmal AF)	1.35	1.03	1.76	0.0269*
Hypertension	1.10	0.83	1.45	0.519
Diabetes	1.00	0.75	1.33	0.987
History of heart failure	2.10	1.62	2.74	<.0001*
History of a stroke/TIA	0.79	0.53	1.18	0.255
History of AF ablation	0.96	0.58	1.60	0.883
DOAC use (vs. warfarin)	1.14	0.89	1.46	0.305
Antiplatelet use	1.78	1.35	2.36	<.0001*
**Stroke/SE**				
CrCl <50 mL/min (vs. CrCl ≥80mL/min)	2.13	1.14	4.00	0.0182*
Age ≥75 years	1.39	0.93	2.09	0.109
Female sex	0.80	0.51	1.24	0.310
Body weight (<50 kg)	1.42	0.84	2.40	0.187
Paroxysmal AF (vs. non-paroxysmal AF)	0.81	0.55	1.19	0.287
Hypertension	1.37	0.89	2.09	0.149
Diabetes	1.03	0.68	1.55	0.896
History of heart failure	0.81	0.52	1.25	0.338
History of a stroke/TIA	2.17	1.44	3.27	0.0002*
History of AF ablation	0.73	0.33	1.61	0.435
DOAC use (vs. warfarin)	1.26	0.89	1.79	0.193
Antiplatelet use	0.98	0.63	1.54	0.940
**Major bleeding**				
CrCl <50 mL/min (vs. CrCl ≥80mL/min)	1.83	1.02	3.29	0.0434*
Age ≥75 years	1.22	0.80	1.87	0.354
Female sex	0.77	0.48	1.23	0.269
Body weight (<50 kg)	0.92	0.50	1.70	0.796
Paroxysmal AF (vs. non-paroxysmal AF)	0.96	0.65	1.42	0.842
Hypertension	1.34	0.87	2.08	0.187
Diabetes	1.24	0.83	1.85	0.292
History of heart failure	0.80	0.51	1.27	0.348
History of a stroke/TIA	0.95	0.55	1.64	0.851
History of AF ablation	0.48	0.19	1.21	0.122
DOAC use (vs. warfarin)	0.96	0.67	1.38	0.833
Antiplatelet use	1.20	0.77	1.87	0.414

AF=atrial fibrillation; CI=confidence intervals; CrCl=creatinine clearance; DOAC=direct oral anticoagulant; HR=hazard risk; SE=systemic embolism. * means p <0.05.

Table S2. Multivariable analysis of the risk factors for each adverse clinical event by using continuous variables for all explanatory factors.

Outcomes	Adjusted HR	95% CI min	95% CI max	p-Value
**Death**				
Log CrCl	0.49	0.31	0.76	0.0017*
Age (years)	1.08	1.06	1.11	<.0001*
Female sex	0.51	0.35	0.74	0.0004*
Body weight (kg)	0.99	0.97	1.01	0.313
Paroxysmal AF (vs. non-paroxysmal AF)	0.84	0.60	1.16	0.285
Hypertension	0.73	0.54	1.00	0.0479*
Diabetes	1.28	0.92	1.76	0.139
History of heart failure	1.60	1.18	2.18	0.0027*
History of stroke/TIA	0.98	0.64	1.50	0.930
History of AF ablation	0.85	0.39	1.87	0.686
DOAC use (vs. warfarin)	1.05	0.78	1.40	0.761
Antiplatelet use	1.22	0.87	1.71	0.256
**Cardiovascular events**				
Log CrCl	0.41	0.29	0.58	<.0001*
Age (years)	1.04	1.02	1.06	<.0001*
Female sex	0.86	0.63	1.18	0.341
Body weight (kg)	1.01	1.00	1.03	0.057
Paroxysmal AF (vs. non-paroxysmal AF)	1.37	1.05	1.78	0.0199*
Hypertension	1.01	0.76	1.34	0.969
Diabetes	0.98	0.73	1.31	0.880
History of heart failure	2.04	1.56	2.66	<.0001*
History of stroke/TIA	0.80	0.54	1.19	0.271
History of AF ablation	1.03	0.62	1.71	0.916
DOAC use (vs. warfarin)	1.20	0.93	1.54	0.154
Antiplatelet use	1.75	1.33	2.32	<.0001*
**Stroke/SE**				
Log CrCl	0.50	0.28	0.88	0.0171*
Age (years)	1.03	1.00	1.05	0.064
Female sex	0.89	0.57	1.38	0.593
Body weight (kg)	1.00	0.98	1.02	0.729
Paroxysmal AF (vs. non-paroxysmal AF)	0.82	0.56	1.21	0.321
Hypertension	1.32	0.86	2.02	0.203
Diabetes	1.01	0.67	1.52	0.968
History of heart failure	0.80	0.51	1.26	0.339
History of stroke/TIA	2.16	1.43	3.25	0.0002*
History of AF ablation	0.73	0.33	1.62	0.444
DOAC use (vs. warfarin)	1.31	0.92	1.86	0.137
Antiplatelet use	0.96	0.61	1.51	0.865
**Major bleeding**				
Log CrCl	0.43	0.25	0.75	0.0029*
Age (years)	1.02	1.00	1.05	0.110
Female sex	0.90	0.56	1.45	0.661
Body weight (kg)	1.02	1.00	1.04	0.0180*
Paroxysmal AF (vs. non-paroxysmal AF)	0.98	0.66	1.45	0.920
Hypertension	1.24	0.80	1.93	0.343
Diabetes	1.17	0.78	1.75	0.446
History of heart failure	0.81	0.51	1.28	0.365
History of stroke/TIA	0.93	0.54	1.61	0.792
History of AF ablation	0.51	0.20	1.28	0.149
DOAC use (vs. warfarin)	0.99	0.69	1.43	0.976
Antiplatelet use	1.17	0.75	1.83	0.478

AF=atrial fibrillation; CI=confidence intervals; CrCl=creatinine clearance; DOAC=direct oral anticoagulant; HR=hazard risk; SE=systemic embolism.* means p <0.05.
